# Hierarchical discrete representations for coarse-to-fine protein conformation generation

**DOI:** 10.1093/bioinformatics/btag611

**Published:** 2026-08-14

**Authors:** SeokJun On, Yujin Jeong, Kang-Hyeon Kim, Kyungheon Kang, Eun-Sol Kim

**Affiliations:** Department of Artificial Intelligence, Hanyang University, Seoul 04763, Republic of Korea; Department of Artificial Intelligence, Hanyang University, Seoul 04763, Republic of Korea; Department of Artificial Intelligence, Hanyang University, Seoul 04763, Republic of Korea; Department of Artificial Intelligence, Hanyang University, Seoul 04763, Republic of Korea; Department of Artificial Intelligence, Hanyang University, Seoul 04763, Republic of Korea; Department of Computer Science, Hanyang University, Seoul 04763, Republic of Korea

## Abstract

**Motivation:**

Protein conformation generation remains a fundamental challenge in structural biology and machine learning. Proteins are highly dynamic macromolecules, and their functions are governed not only by static 3D structures but also by their conformational flexibility. Therefore, generating a diverse ensemble of physically plausible structures is crucial for modeling conformational heterogeneity, characterizing intrinsically disordered proteins (IDPs), and supporting structure-based drug discovery. However, existing generative models often operate in continuous coordinate space. This approach makes it difficult to impose structural priors or handle complex, long-range dependencies, frequently leading to a significant trade-off where models must sacrifice structural validity to ensure conformational diversity.

**Results:**

To overcome these limitations, we propose a novel approach that learns hierarchical discrete representations of protein structures using vector quantization. Inspired by the success of VQ-VAE models in vision, we discretize residue-level structural contexts into learnable codebooks at multiple levels of granularity. Our framework first constructs a coarse-grained scaffold that captures global topology and secondary structures, and subsequently conditions on this scaffold to progressively refine the fine-grained local geometry. This coarse-to-fine generation mechanism enables both efficient sampling and highly accurate reconstruction. Through extensive experiments, we demonstrate that our method outperforms state-of-the-art models such as ESMDiff across challenging benchmark datasets, including BPTI MD trajectories and conformational-changing pairs (apo/holo and fold-switch). Our model mitigates the trade-off between conformational diversity and structural validity, generating realistic ensembles while providing interpretable and scalable representations of protein geometry. Ultimately, this work offers a powerful new paradigm for flexible biomolecular modeling.

**Availability and implementation:**

The code is available at https://github.com/vanha9/hierarchical_conformation_generation. Archived at https://zenodo.org/records/21817083.

## 1 Introduction

Biological functions are governed not only by static 3D structures but fundamentally by their conformational flexibility. Therefore, generating an ensemble of physically plausible structures is crucial for drug discovery tasks, including target identification and docking. However, current models are limited to predicting a single static structure, failing to capture intrinsically disordered proteins or transition states. While molecular dynamics (MD) simulations can theoretically address these dynamics, they remain computationally prohibitive. Consequently, diffusion models have emerged as a promising paradigm for directly modeling these flexible protein conformations.

Existing diffusion-based generative models primarily adopt a paradigm of generating protein structures at a single resolution (Jing *et al.* 2023, Lu *et al.* 2024a,b). However, proteins inherently possess a structural hierarchy where coarse-grained characteristics, such as global topology and secondary structures, coexist with fine-grained details that determine structure at the residue and atomic levels. Single-resolution approaches are required to learn these distinct layers of information simultaneously within a unified dimension, without explicit separation. Consequently, they fail to effectively capture features across different scales. As a result, the models face a significant trade-off, which is particularly pronounced for large proteins: generated structures often fail to properly form secondary structures, or the models must excessively sacrifice diversity to ensure structural validity.

To overcome this limitation, we propose a new paradigm, coarse-to-fine protein conformation generation, which reflects the structural hierarchy of proteins. Our model first constructs coarse features that capture the global topology and secondary structures, and then uses this structural scaffold as a condition to progressively refine the residue-level fine-grained structure. This the hierarchical approach decomposes the complex dependencies of protein structures, allowing the model to first capture rigid components (e.g. secondary structures) and then accurately sample the diverse conformations of flexible regions connecting them. Notably, coarse tokens are designed to preserve topological information, ensuring physically valid structure generation, while effectively encoding the physical flexibility of amino acids. Leveraging this dual capability, we can modulate the flexibility of the coarse scaffold itself via top-*k* sampling. This approach enables us to overcome the limitations of single-resolution prediction and successfully capture the diverse conformational ensembles inherent to real proteins.

**Figure 1:**
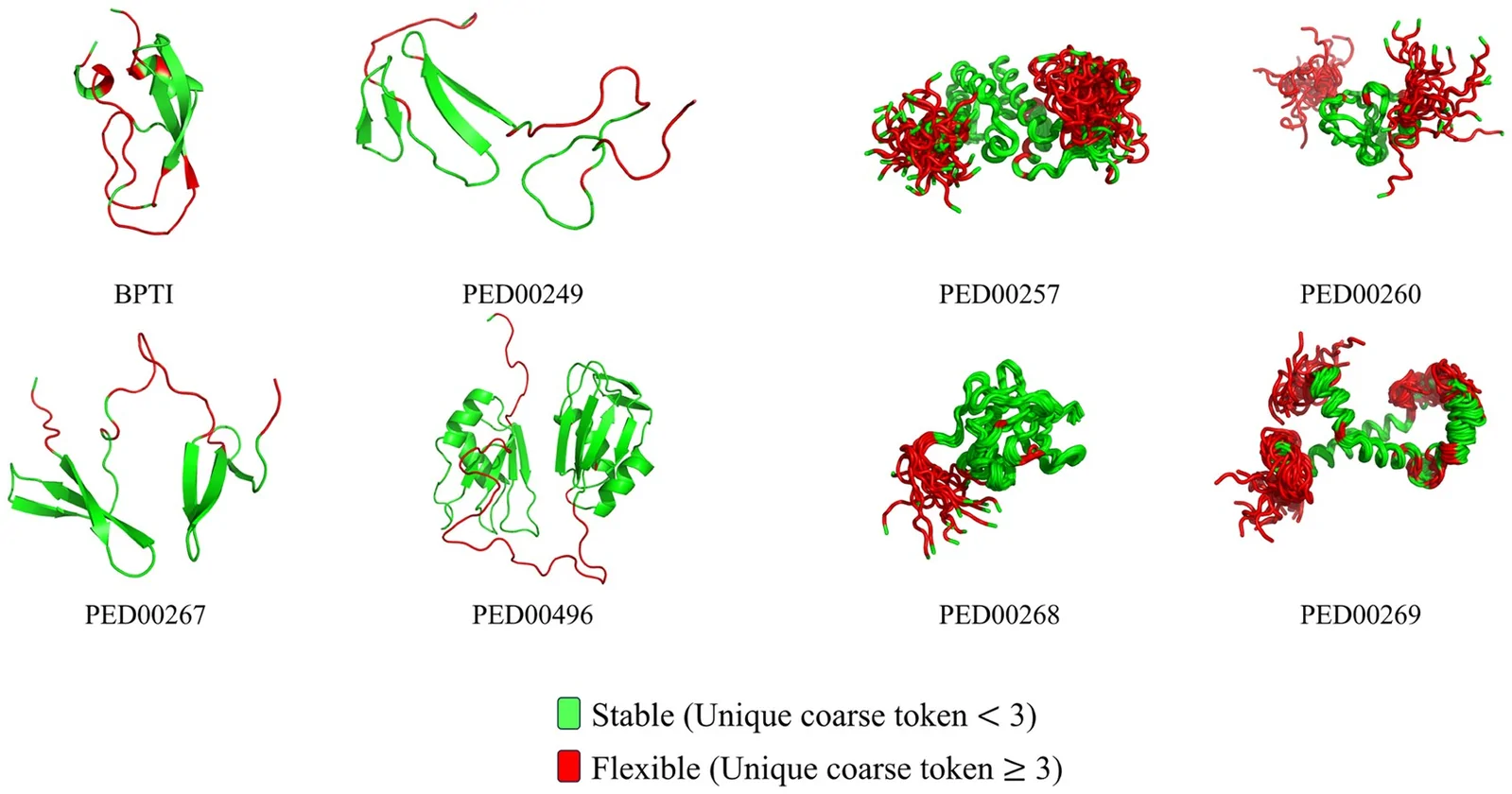
Visualization of coarse token variability. Structures are colored based on the number of unique tokens assigned across five ground-truth conformers. Green regions (<3 unique types) correspond to rigid structures, while red regions (≥3 unique types) align with flexible areas such as loops.

**Figure 2:**
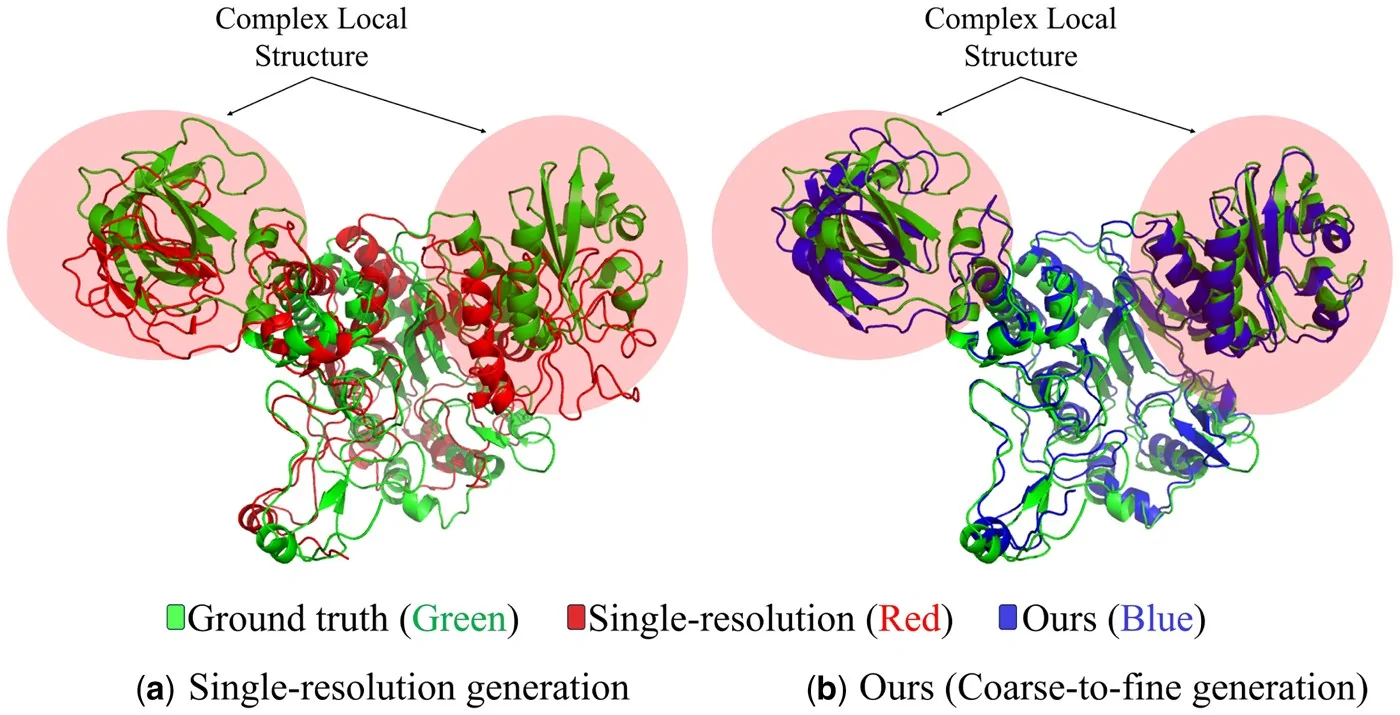
Comparison of structural fidelity in challenging areas. Highlighted complex local structures (circled regions) illustrate the performance gap. (a) The single-resolution generation model fails to reconstruct these intricate geometries, resulting in misalignment with the ground truth structure. (b) Our method successfully recovers the native topology and aligns precisely with the ground truth structure.

The main contributions of our work are as follows:

- *Hierarchical generative framework*: We propose a novel coarse-to-fine generative model that progressively refines protein structures. By decomposing the generation process, the hierarchical approach effectively models the complex dependencies between global folding patterns and local geometric details.
- *Controllable diversity via token manipulation*: We validate that the coarse token diversity correlates with structural flexibility as shown in Fig. 1. Leveraging this insight, we employ top-*k* sampling on coarse tokens to generate diverse scaffolds, reflecting the intrinsic dynamics of proteins beyond static prediction.
- *Structurally robust sampling*: Unlike single-resolution approaches, our model first establishes a rigid structural scaffold (e.g. secondary structures) before progressively refining it. As shown in Fig. 2, this mechanism ensures physical validity while sampling diverse conformations, successfully balancing structural realism with flexibility.

## 2 Related works

### 2.1 Protein structure prediction and ensemble generation

Following the success of AlphaFold2 (Jumper *et al.* 2021), research in protein structure prediction has expanded beyond predicting single, static structures to generating diverse conformational ensembles that reflect the dynamic properties of proteins. To sample functionally important structural states, early approaches utilized MSA subsampling or clustering (del Alamo *et al.* 2022, Bryant and Noé 2024) to induce structural variations. However, these methods are fundamentally constrained by the deterministic nature of the base predictors and incur high computational costs. More recent approaches leverage deep learning generative models such as diffusion probabilistic models and flow matching (Jing *et al.* 2023, 2024, Lu *et al.* 2024b).

However, existing end-to-end generation methods struggle to explicitly learn the complex interdependencies between the global fold and local details, particularly when modeling long-range interactions in large proteins. This limitation often leads to structural hallucinations or the accumulation of errors during the generation process. To address this, our work proposes a new paradigm that hierarchically decomposes structure generation and progressively refines the structure in an autoregressive manner. Our model first generates coarse fold information and then conditions on it, utilizing it as a robust structural scaffold, to stably generate fine-grained structures. This approach enables an effective exploration of physically plausible conformational ensembles while enhancing generative stability.

### 2.2 Discrete representation of protein structures

Representing protein structures as discrete token sequences, rather than continuous 3D coordinates, is an active area of research (Liu *et al.* 2024, Gao *et al.* 2025, Pourmirzaei *et al.* 2025) that allows for the application of powerful natural language processing models by treating structures as a “language.” Notably, ESM3 (Hayes *et al.* 2025) utilizes a VQ-VAE (Van Den Oord and Vinyals 2017) to tokenize local structural motifs into a structural alphabet, using them as prediction targets for a language model to achieve high performance. However, most existing tokenization methods employ a single-resolution codebook, which limits their ability to effectively capture both global fold information and local geometric details simultaneously. Our work models multi-scale structural information—from coarse-to-fine levels—by employing hierarchical tokenization with codebooks of varying sizes.

### 2.3 Hierarchical generation models

When generating complex, high-dimensional data, a hierarchical (coarse-to-fine) strategy that first establishes a low-resolution overview and then progressively adds details is highly effective. In image generation, autoregressive models like VQ-VAE-2 (Razavi *et al.* 2019) and the Visual Auto-Regressive (VAR) model (Tian *et al.* 2024) have demonstrated the efficacy of this strategy by first generating a low-resolution image and then sequentially generating a high-resolution image conditioned on it. Inspired by these successes, our work adapts this hierarchical approach to the discrete token space of protein structures. This enables our model to explicitly capture global dependencies and stably generate a diverse ensemble of physically plausible conformations.

## 3 Method

In this section, we introduce Hierarchical Protein Conformation Generation, a novel hierarchical approach for protein conformation generation. Our proposed method utilizes a VQ-VAE-based tokenizer (Van Den Oord and Vinyals 2017) to represent protein structures at multiple resolutions and an autoregressive generative model to generate diverse structural tokens from an amino acid sequence in a coarse-to-fine manner.

### 3.1 Hierarchical protein structure tokenization

In this work, we tokenize the 3D structure of a protein by encoding the local structural environment of each amino acid. Let *X* denote the input protein structure consisting of *N* residues. For a central residue, the encoder *E* generates a continuous latent feature vector representing its structural characteristics based on the relative positions of its *k*-nearest neighboring amino acids. A key principle of this method is that residues that are contiguous in sequence or in contact due to folding share similar local environments, causing their encoded vectors to be proximate in the latent space. To capture structural information at multiple resolutions, we employed a hierarchical VQ-VAE with a set of learnable codebooks.


$$Z_{\left(l\right)}=\{z_{\left(l\right)}^{1},z_{\left(l\right)}^{2},\ldots ,z_{\left(l\right)}^{K_{l}}\},\;l\in \{\text{coarse},\;\text{mid},\;\text{fine}\}$$

*l* denotes the hierarchy level and $K_{l}$ represents the codebook size. In this study, we empirically set the codebook sizes as $\left(K_{\text{coarse}},K_{\text{mid}},K_{\text{fine}})=(32,512,4096\right)$. The encoder maps the input structure *X* to continuous latent representations e=E(X). Subsequently, these continuous vectors are quantized into discrete tokens via nearest neighbor search in the codebook space. Specifically, for each residue position i∈{1,2,…,N}, the discrete token index $s_{\left(l\right)}^{i}$ and the corresponding quantized vector $z_{\text{quant}(l)}^{i}$ are obtained as follows:


(1) $$s_{\left(l\right)}^{i}=\underset{k\in \{1,\ldots ,K_{l}\}}{*\mathrm{argmin}}\|e^{i}-z_{\left(l\right)}^{k}\|,\;z_{\text{quant}(l)}^{i}=z_{\left(l\right)}^{s_{\left(l\right)}^{i}}$$

**Figure 3:**
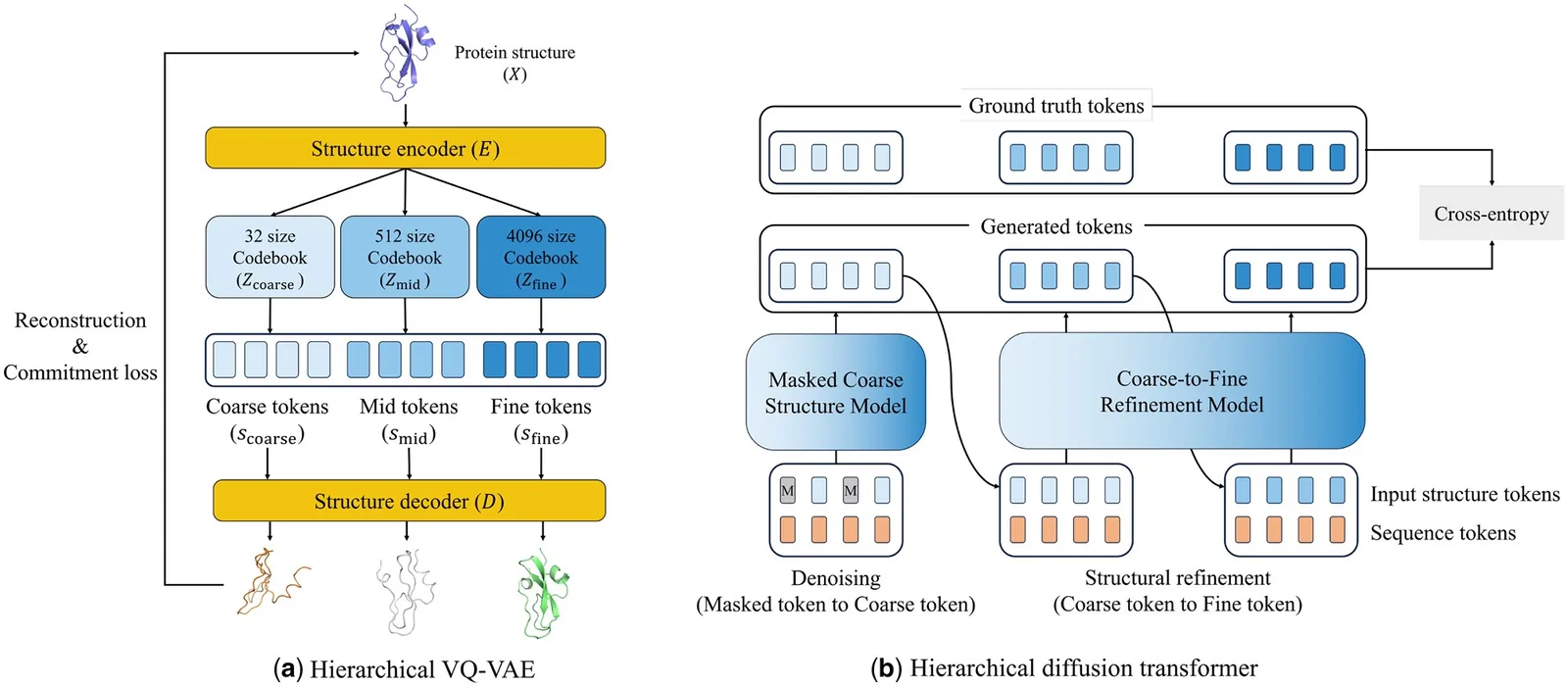
(a) Hierarchical tokenization using VQ-VAE: The protein structure is discretely tokenized into coarse-to-fine representations using codebooks of different sizes. The tokenizer is optimized via structural reconstruction and commitment losses. (b) Coarse-to-fine diffusion transformer: Conditioned on amino acid sequences, the model generates coarse tokens from masked inputs (M) via discrete diffusion, followed by the autoregressive prediction of fine-grained tokens. Finally, the predicted tokens are passed through the structure decoder to generate the 3D structure.

As illustrated in the tokenization process of Fig. 3a, we extract hierarchical structural information by utilizing codebooks of different sizes ($K_{\text{coarse}}<K_{\text{mid}}<K_{\text{fine}}$).

- A small codebook tends to map similar vectors to the same token. Consequently, structurally adjacent residues are often represented by the same tokens, yielding a token sequence that captures coarse-grained information such as secondary structures and domains. Furthermore, this approach effectively abstracts minor geometric variations by mapping them to the same coarse token, while assigning distinct tokens to significantly different structures. This distinction provides enriched information regarding the similarity and divergence of local structural environments.
- Conversely, a large codebook maps similar vectors to distinct, yet finely differentiated tokens. This preserves the unique geometric features of each residue even if they are structurally close, generating a token sequence that contains more detailed, fine-grained information.

Ultimately, this process yields a set of multi-resolution token sequences, $\left\{s_{\text{coarse}},s_{\text{mid}},s_{\text{fine}}\right\}$ for a single protein structure, each representing a different level of structural detail. Finally, the quantized representations from all levels are fed into the structure decoder *D* to reconstruct the original protein structure ${\hat{X}}_{l}=D(z_{\text{quant}(l)})$. The entire VQ-VAE framework is trained end-to-end by minimizing the total loss, which is the sum of losses calculated at each level l∈{coarse,mid,fine}. As described above, the coarse tokens, quantized by a smaller codebook, learn to abstract broad structural topologies, while the fine tokens leverage a larger codebook to preserve intricate geometric details. This joint optimization enables the model to effectively learn the interrelationships between tokens at different levels, allowing a single structural representation to be captured through both abstract coarse tokens and detailed fine tokens.


(2) $$L_{l}=\underset{L_{\text{recon}}}{\underset{︸}{\|X-{\hat{X}}_{l}{\|}^{2}}}+\sum_{i}\underset{L_{\text{commit}}}{\underset{︸}{\|e^{i}-\mathrm{sg}[z_{\text{quant}(l)}^{i}]{\|}^{2}}}$$

where sg[·] denotes the stop-gradient operator.

To verify whether the coarse tokens effectively capture the protein dynamics, we tokenized the ground-truth ensemble structures and performed an analysis. Further details can be found in Experiments section.

### 3.2 Hierarchical generative learning

We employ a coarse-to-fine generative framework to synthesize protein structures from amino acid sequences. This process first establishes a coarse-grained global fold via diffusion, and subsequently refines it into detailed geometry using an autoregressive approach as shown in Fig. 3b.

#### 3.2.1 Stage 1: coarse token generation via discrete diffusion

The first stage constructs the structural scaffold using coarse-grained tokens. The model takes the amino acid sequence tokens and a fully masked initial structural token sequence as inputs. These input tokens are transformed into embedding vectors and then summed before being fed into a language model. Subsequently, the model generates the final coarse structure token sequence, $s_{\text{coarse}}$, through a multi-step denoising process (Austin *et al.* 2023). This diffusion-based approach not only demonstrates effective performance on the complex task of generating the global fold from a sequence but also allows for the generation of diverse coarse structural tokens for the same input, owing to the stochastic nature of the denoising process. This contributes to modeling the various conformational states and inherent flexibility of proteins.

### 3.2.2 Stage 2: autoregressive refinement

Once the coarse structure tokens are generated, the model refines this scaffold into high-resolution structure in an autoregressive manner. The model predicts the subsequent fine-grained tokens (e.g. from coarse to mid, and mid to fine) by taking the generated coarse structure tokens and the amino acid sequence as combined input. In this process, residues mapped to the same coarse token provide a context of structural similarity, while the subsequent prediction of distinct fine tokens enables the reconstruction of their specific geometric nuances. Essentially, this refines structural details within the framework of the established coarse fold.

This coarse-to-fine strategy significantly reduces generation uncertainty at each resolution level. By utilizing coarse structural information as an explicit condition for predicting complex fine-grained details, the model effectively learns interscale correlations and suppresses error propagation compared to single-step generation.

## 3.2.3 Diversity via top-*k* sampling strategy

During inference, we employ a stochastic autoregressive sampling with top-*k* truncation followed by manual re-weighting of the selection probabilities into fixed ratios to specifically promote conformational diversity within the generated ensemble. Unlike single-resolution models where selecting non-optimal tokens often leads to physically invalid or fragmented structures, our coarse-to-fine paradigm effectively decouples diversity from structural validity.

We first utilize top-*k* sampling in the coarse stage to generate diverse global scaffold candidates. Our empirical analysis identifies this stage as the optimal locus for stochasticity: unlike the fine stage where stochastic sampling noise often persists as uncorrected structural errors, coarse-level perturbations are effectively rectified by the subsequent fine-token prediction module. The fine-token prediction module conditions on these scaffolds to reconstruct detailed geometries. This mechanism ensures that even diverse, less probable coarse trajectories are successfully refined into physically plausible structures. Consequently, our model generates a realistic yet diverse ensemble, overcoming the trade-off between diversity and structural integrity often observed in direct single-resolution sampling.

## 4 Experiments

To evaluate the effectiveness of our proposed method, we set up comparative baselines following experimental protocols established in prior studies. These include MSA-subsampling (del Alamo *et al.* 2022), EigenFold (Jing *et al.* 2023), AlphaFlow, and ESMFlow (Jing *et al.* 2024).

For models involving distinct sampling or modeling schemes, we explicitly specify the variants used for a comprehensive comparison: Str2Str (Lu *et al.* 2024b) with Probability Flow (PF) and Stochastic Differential Equation (SDE) solvers, and ESMDiff (Lu *et al.* 2024a) configured for Iterative Decoding (ID) and Denoising Diffusion Probabilistic Modeling (DDPM).

To ensure a fair and direct comparison with state-of-the-art baselines like ESMDiff, we intentionally controlled our overall architecture size to match theirs, eliminating unfair advantages from simply scaling up the model. Furthermore, specific hyperparameters were carefully determined to balance generative expressiveness and learning efficiency: the codebook size was selected to capture fine-grained structural details while preventing codebook collapse, and the *k* value in our top-*k* sampling was chosen to optimally balance structural diversity and physical validity by preventing both noise-induced degradation and mode collapse.

The model was trained using static structural data stored in the Protein Data Bank (PDB). To assess its ability to generalize beyond these static snapshots and capture intrinsic protein dynamics, we conducted a comprehensive evaluation using three distinct datasets:

- *BPTI MD trajectories* (Grouazel *et al.* 2006): Used to assess the distribution of physical properties and structural accuracy against ground truth MD simulations.
- *Conformational-changing pairs* (Chakravarty and Porter 2022, Saldaño *et al.* 2022): Utilized to measure the reproducibility of structural variations, including per-residue flexibility and secondary structure transitions.
- *Intrinsically Disordered Proteins (IDPs)* (Ghafouri *et al.* 2024): Employed to validate the model’s capacity to reproduce the physical properties of highly flexible structural ensembles.

Detailed definitions and descriptions of the evaluation metrics used for each dataset are provided in Supplementary Section B, available as supplementary data at *Bioinformatics* online.

To analyze the impact of hierarchical refinement, we considered a 2-stage variant that bypasses the mid-level refinement phase of the proposed 3-stage model. Our primary results are obtained using greedy sampling (i.e. top-1) and a top-3 sampling at three specific ratios. Results for our model reported in the tables correspond to the best-performing configuration among the evaluated sampling strategies for each model. Detailed dataset information and hyperparameter analyses are provided in Supplementary Sections A and C, available as supplementary data at *Bioinformatics* online, respectively.

### 4.1 Validation of coarse tokenization and structural variability

Prior to evaluating the generation performance, we validated whether our coarse quantization with a compact codebook ($|Z_{\text{coarse}}|=32$) accurately reflects the intrinsic dynamics of proteins. While utilizing a small codebook significantly reduces prediction complexity and ensures high accuracy in global fold generation, it raises the question of whether such a compressed representation can sufficiently encode structural diversity.

To address this, we randomly sampled five snapshots from the ground truth conformational ensemble of various proteins. We then encoded each structure into coarse tokens and measured the number of unique token types assigned to each residue position. We hypothesized that a semantically meaningful tokenizer should assign consistent tokens to rigid structural elements while exhibiting variability in flexible regions. As illustrated in Fig. 1, our analysis confirms this correlation. Structurally stable elements, such as alpha-helices and beta-sheets, maintained consistent assignments, typically varying by fewer than three distinct token types. In contrast, physically dynamic regions, including loops and termini, displayed high symbolic diversity, frequently mapping to three or more distinct token types at the same position.

This demonstrates that our coarse tokenizer does not merely memorize coordinates but possesses the structural sensitivity to distinguish between stable cores and dynamic fluctuations, effectively translating physical flexibility into symbolic diversity.

### 4.2 Evaluation on BPTI molecular dynamics

To evaluate the dynamic structural generation performance, we utilized BPTI MD trajectories (Shaw *et al.* 2010), which provide a comprehensive view of the protein’s conformational landscape. We assessed the model’s ability to effectively capture the five distinct kinetic clusters defined within this trajectory (Wang *et al.* 2024) and evaluated its distributional alignment with the ground truth MD simulation data.

**Figure 4:**
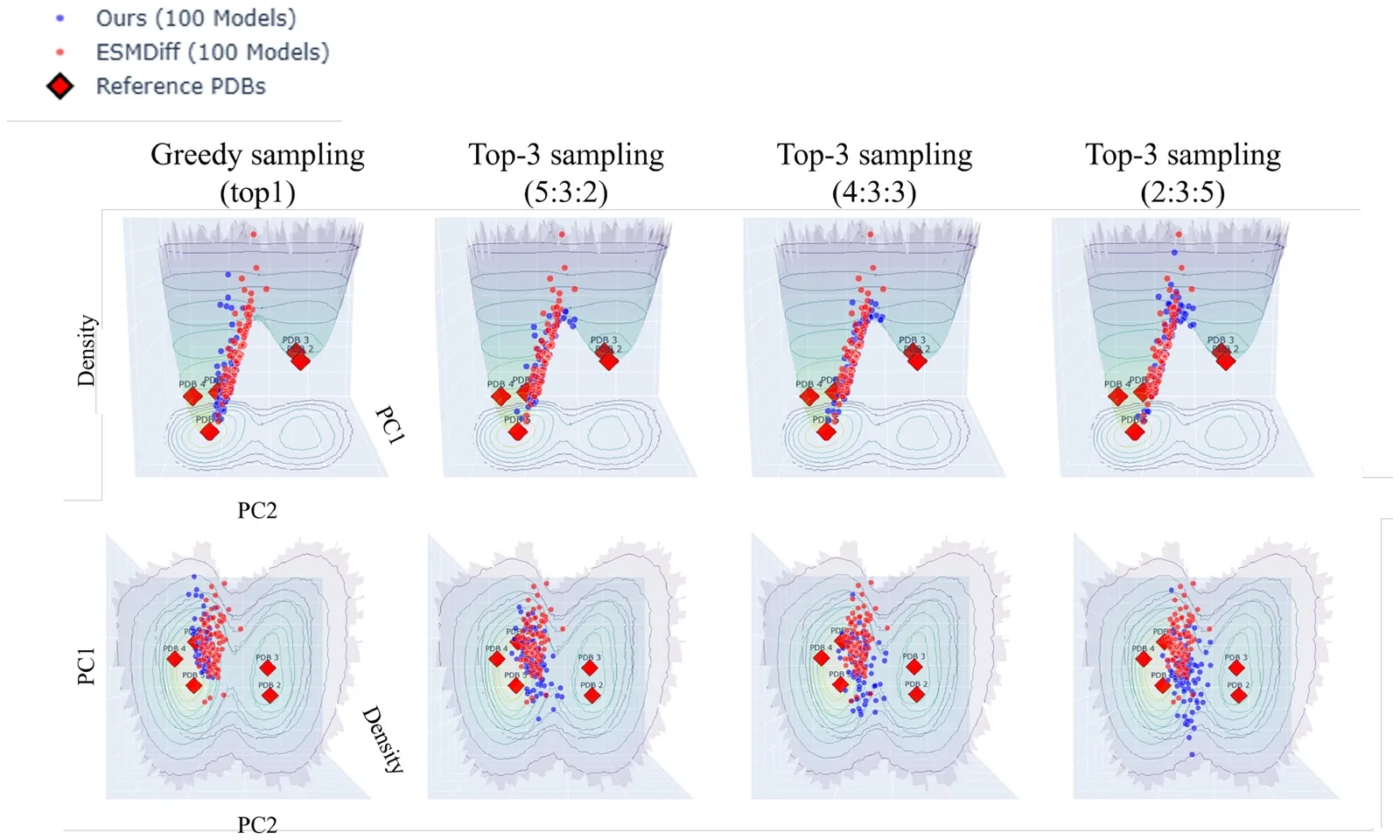
PCA projection of generated structures onto the MD density landscape. Blue and red dots represent our model and ESMDiff (Lu *et al*. 2024a), respectively. The columns compare Greedy sampling with top-3 sampling strategy across different token selection ratios (from 5:3:2 to 2:3:5).

Table 1 and Fig. 4 highlight the distinct advantages of our sampling strategies. Greedy sampling precisely targets the thermodynamically stable clusters 1, 4, and 5, demonstrating high fidelity in reconstructing high-density regions. Crucially, employing top-*k* sampling significantly improves the recovery of the metastable clusters 2 and 3, which correspond to rare transition states often missed by standard generative approaches. This confirms that our framework allows for controllable exploration of both stable cores and diverse transition pathways.

**Table 1:** Performance evaluation on BPTI kinetic clusters.^a^

	${\text{RMSD}}_{C1}$	${\text{RMSD}}_{C2}$	${\text{RMSD}}_{C3}$	${\text{RMSD}}_{C4}$	${\text{RMSD}}_{C5}$
MSA-Subs.	0.921	1.745	2.519	1.688	0.989
AlphaFlow	0.939	1.683	2.394	1.401	0.980
ESMFlow	0.915	1.694	2.424	1.391	0.988
Str2Str (PF)	2.092	2.082	2.774	2.310	2.078
Str2Str (SDE)	2.607	2.457	3.068	2.767	2.431
EigenFold	0.829	1.676	2.470	1.436	1.009
ESMDiff (ID)	0.928	1.634	2.278	1.507	1.010
ESMDiff (DDPM)	0.939	1.620	2.208	1.405	1.001
Ours (3-stage)	0.828	1.622	**2.083**	1.430	0.923
Ours (2-stage)	**0.798**	**1.571**	2.130	**1.345**	**0.888**

a For each of the five kinetic clusters, we evaluated the performance by measuring the RMSD of the closest generated structure. Lower RMSD values indicate improved performance (↓). **Bold** and underlined values represent the best and second-best performance for each metric within this table, respectively. Cluster C3 is particularly challenging to reproduce (Wang *et al.* 2024).

We further assessed physical realism by computing the Jensen–Shannon (JS) divergence against the reference MD trajectories for key structural descriptors: pairwise distances (PwD), time-lagged independent components (TIC), and radius of gyration (RG). Additionally, we used TM-ens and RMSD-ens as metrics to evaluate the ensemble fidelity, along with Validity to measure the fraction of clash-free conformations. The results in Table 2 demonstrate that our model yields significantly lower JS divergence than baselines, confirming that the generated ensemble faithfully reproduces the ground truth thermodynamic landscape.

**Table 2:** Performance evaluation on BPTI molecular dynamics (MD) trajectory.^a^

Method	Distribution divergence			Structural fidelity		
	JS-PwD	JS-TIC	JS-RG	Validity	TM-ens	RMSD-ens
MSA-Subs.	0.615	0.499	0.730	0.990	0.835	1.576
AlphaFlow	0.474	0.499	0.730	**1.000**	0.843	1.479
ESMFlow	0.488	0.487	0.734	0.980	0.839	1.482
Str2Str (PF)	0.503	0.513	0.326	0.910	0.705	2.267
Str2Str (SDE)	0.531	0.570	0.394	0.860	0.651	2.666
EigenFold	0.512	0.487	0.768	0.660	0.839	1.482
ESMDiff (ID)	0.378	0.358	0.452	0.950	0.844	1.471
ESMDiff (DDPM)	0.346	0.341	0.356	0.940	0.845	1.435
Ours (3-stage)	**0.330**	0.370	0.153	0.960	0.842	1.447
Ours (2-stage)	0.335	**0.281**	**0.107**	**1.000**	**0.850**	**1.409**

a Jensen–Shannon (JS) divergence of the PwD, TIC (Pérez-Hernández *et al.* 2013), and RG distributions, alongside TM-ens and RMSD-ens, computed for the generated BPTI conformational ensemble. Lower Distribution Divergence and RMSD-ens (↓), and higher TM-ens and validity, indicate better performance (↑). **Bold **and underlined values represent the best and second-best performance for each metric within this table, respectively.

### 4.3 Evaluation on conformational-changing pairs

**Figure 5:**
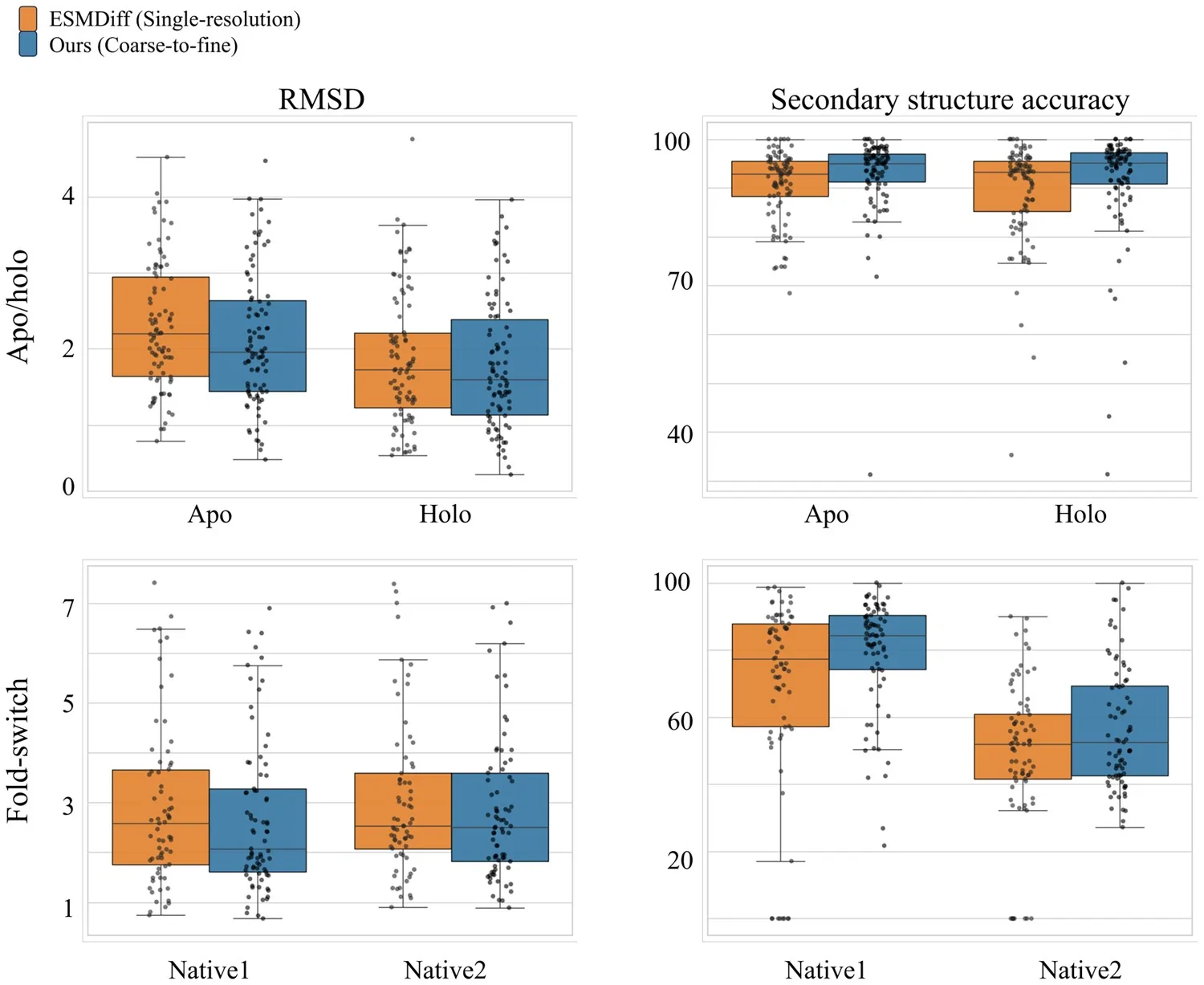
Evaluation of structural accuracy on conformational-changing pairs. Comparison of RMSD (left panel) and secondary structure accuracy (right panel) between the single-resolution baseline and our model on the conformational-changing pair dataset. In each subplot, the left and right bars represent the baseline and our model, respectively.

To validate the structural realism and fine-grained fidelity of the conformations generated for modeling structural transitions, we utilized the 87 apo/holo pairs (Saldaño *et al.* 2022) and 69 fold-switch pairs (Chakravarty and Porter 2022), excluding all sequences used during training. Consistent with established benchmarks, we constructed an ensemble by sampling five conformations per target. As shown in Table 3, our method outperformed the single-resolution baseline in terms of residual flexibility; while not surpassing the top-performing baseline, the results remain competitive. While prior works primarily focused on residue flexibility correlations, we posit that accurately capturing the relative rigidity of local secondary structures is a prerequisite for modeling such flexibility while maintaining structural validity. Since these rigid elements serve as stable geometric anchors during structural variations, their precise reconstruction is essential. To evaluate secondary structure conservation, we assigned secondary structure labels to both the generated samples and the ground truth using the DSSP algorithm (Kabsch and Sander 1983), calculating accuracy based on residue-level agreement. The results in Fig. 5 confirm that the coarse-to-fine strategy effectively preserves global structural topology, as evidenced by the low RMSD. Furthermore, the high secondary structure accuracy suggests that the model successfully reconstructs rigid local geometries by correctly forming the underlying hydrogen bond networks of alpha-helices and beta-sheets.

**Table 3:** Performance evaluation on conformational-changing pairs.^a^

Method	Apo/holo			Fold-switch		
	ResFlex gl.	ResFlex pt.	TM-ens	ResFlex gl.	ResFlex pt.	TM-ens
MSA-Subs.	0.445	0.422/0.382	**0.863**/**0.895**	0.262	0.304/0.318	**0.739**/**0.814**
AlphaFlow	**0.563**	**0.533**/**0.565**	0.862/0.885	0.394	**0.381**/**0.373**	0.734/0.812
ESMFlow	0.448	0.506/0.523	0.854/0.889	0.327	0.343/0.351	0.702/0.758
Str2Str (PF)	0.136	0.314/0.326	0.744/0.763	0.150	0.203/0.177	0.598/0.629
Str2Str (SDE)	0.061	0.294/0.284	0.578/0.585	0.048	0.177/0.184	0.431/0.419
EigenFold	0.121	0.404/0.401	0.826/0.869	0.328	0.272/0.222	0.617/0.659
ESMDiff (ID)	0.458	0.488/0.507	0.842/0.877	0.391	0.323/0.352	0.657/0.719
ESMDiff (DDPM)	0.373	0.478/0.488	0.841/0.884	0.408	0.331/0.341	0.635/0.706
Ours (3-stage)	0.486	0.493/0.521	0.844/0.876	**0.453**	0.345/0.355	0.631/0.726
Ours (2-stage)	0.419	0.496/0.514	0.854/0.887	0.447	0.309/0.307	0.643/0.714

a For each of the 87 apo/holo (Saldaño *et al.* 2022) and 69 fold-switch pairs (Chakravarty and Porter, 2022), five structures were sampled to measure residue flexibility and TM-scores. We report the mean and median values for ResFlex pt. and TM-ens. Higher scores indicate improved performance (↑). **Bold** and underlined values represent the best and second-best performance for each metric within this table, respectively.

**Figure 6:**
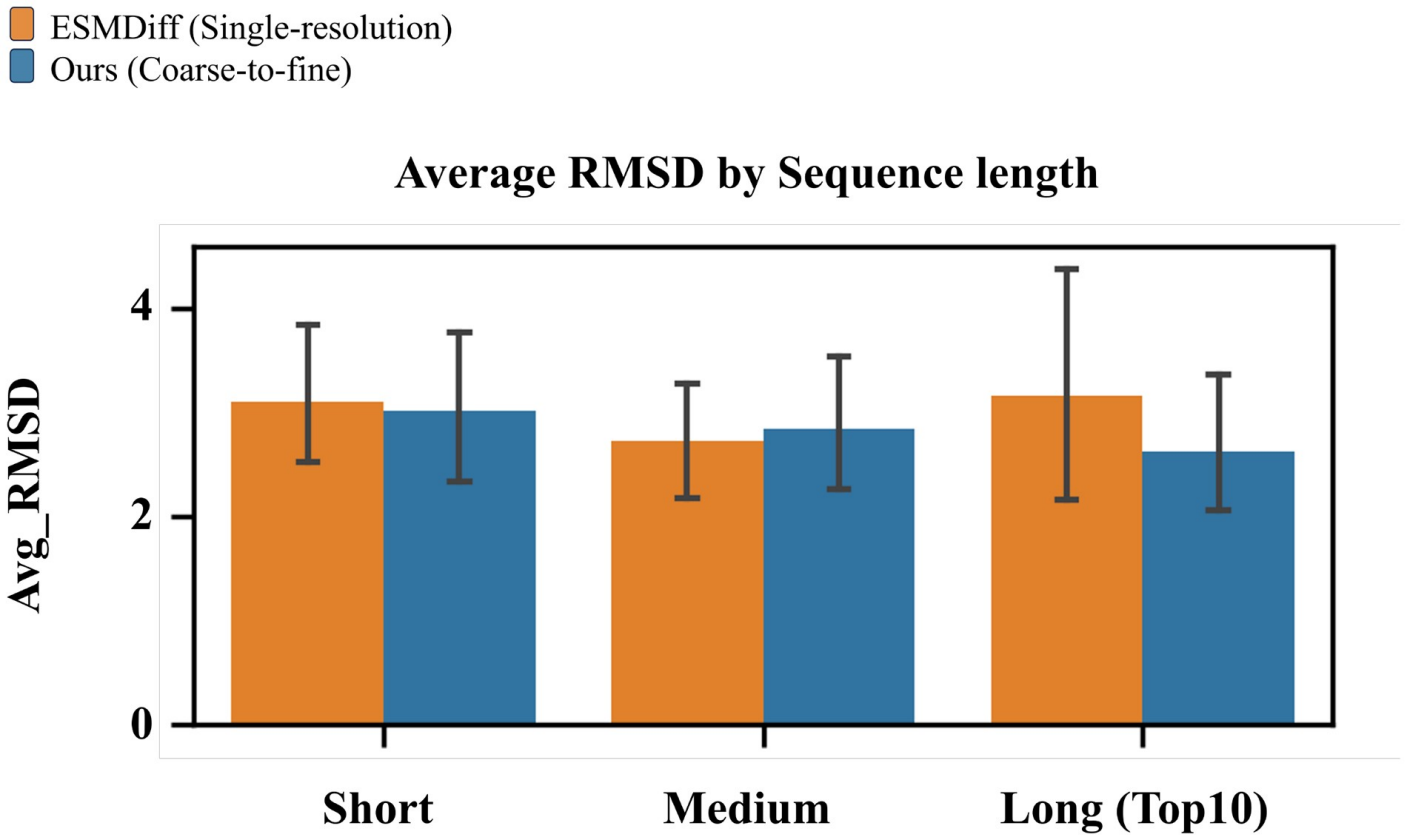
Performance comparison based on sequence length. The plot compares the average RMSD across targets stratified into short, medium, and long (top 10 longest sequences) categories.

As visually highlighted in Fig. 2, our model successfully produces high-quality structures for large proteins that align well with the ground truth, whereas the single-resolution baseline tends to collapse under structural complexity. To quantify this scalability, we categorized the 87 targets into short, medium, and long (top 10) subgroups as shown in Fig. 6. In the Short and Medium categories, the performance of our model was similar to that of the baseline. However, a distinct advantage was observed in the Long group, where our method achieved a comparatively lower RMSD, proving a substantial performance divergence. These results indicate that although existing approaches are effective for short sequences, the hierarchical paradigm is essential for handling the challenges posed by long and complex protein chains.

### 4.4 Analysis on protein Ensemble Database (PED)

Unlike globular proteins, IDPs lack a fixed three-dimensional structure and instead function as dynamic conformational ensembles. While the Protein Data Bank (PDB) (wwPDB consortium 2018) primarily focuses on well-defined globular structures, it offers limited data on IDPs containing extensive disordered regions. To address this limitation, the PED (Ghafouri *et al.* 2024) has been established as a specialized repository that systematically aggregates structural ensemble information for IDPs. In this study, we utilized the PED benchmark to evaluate whether our proposed model can be effectively applied not only to well-folded proteins but also to uncertain structural ensembles characterized by high conformational entropy. Following the experimental protocol of the ESMDiff model (Lu *et al.* 2024a), we curated an evaluation dataset consisting of 110 entries, ensuring the exclusion of any sequences that overlap with the PDB training set.

Given that IDPs are not characterized by a single native structure and exhibit variable ensemble sizes, residue-wise alignment is ill-suited for capturing their structural variability. Accordingly, we employ the MAE of RG, a metric proposed by (Janson *et al.* 2023), to assess whether the generated ensembles match the global size characteristics of the reference ensembles. To further evaluate the generated samples, we compare PwD across all residue pairs and use residue contact maps to examine local contact probabilities.

Our model achieves competitive results across the core evaluation metrics of the PED benchmark. Without relying on multiple sequence alignment (MSA) information, it matches the performance of MSA-Subs (del Alamo *et al.* 2022) in terms of ensemble reconstruction, as shown in Table 4. Compared to the single-resolution model, our approach outperforms it across all metrics, demonstrating that hierarchical generation enables finer-grained sampling of the high-entropy conformational landscape of IDPs.

**Table 4:** Performance evaluation on the Protein Ensemble Database (PED) (Ghafouri *et al.* 2024).^a^

	Pairwise distance	Radius of gyration	Contact map
MSA-Subs.	6.691/4.214	4.245/2.213	**0.178**/**0.112**
AlphaFlow	7.161/**3.553**	4.915/**1.562**	0.231/0.162
ESMFlow	7.622/4.215	5.043/1.592	0.259/0.189
Str2Str (PF)	8.717/5.626	5.280/2.834	0.266/0.192
Str2Str (SDE)	10.222/7.769	6.271/3.796	0.313/0.235
EigenFold	12.268/6.127	9.267/4.632	1.316/0.609
ESMDiff (ID)	6.953/4.945	4.506/2.337	0.289/0.211
ESMDiff (DDPM)	7.085/4.831	4.531/2.204	0.352/0.266
Ours (3-stage)	6.667/4.633	4.093/2.229	0.211/0.144
Ours (2-stage)	**6.493**/4.879	**4.058**/2.198	0.201/0.135

a Ten structures were sampled from each of the 110 protein ensembles to measure the mean absolute error (MAE) of pairwise distance, radius of gyration, and contact map (Janson *et al.* 2023). We report the mean and median values. Lower MAE values indicate improved performance (↓). **Bold **and underlined values represent the best and second-best performance for each metric within this table, respectively.

## 5 Conclusion

In this work, we presented a novel hierarchical generative framework designed to resolve the inherent trade-off between structural stability and conformational diversity in protein modeling. By redefining protein structures not merely as sets of 3D coordinates but as hierarchical semantic entities, we established a process where a coarse topology serves as a guiding scaffold for detailed geometric refinement.

A pivotal contribution of this study lies in the hierarchical framework itself, which enables the model to capture high-level structural semantics. This design philosophy is empirically validated by the semantic interpretability of our coarse tokenizer. Through the analysis of structural variants, we demonstrated that the symbolic diversity of learned coarse tokens directly mirrors the intrinsic physical flexibility of proteins. Specifically, regions with high token variability were mapped to dynamic areas such as loops and termini, while rigid secondary structures maintained consistent token assignments. This confirms that our hierarchical approach effectively encodes biologically meaningful structural dynamics.

We also successfully overcame the stability-diversity trade-off by leveraging these physical characteristics. While existing models typically sacrifice diversity to ensure stability, or compromise structural integrity to pursue diversity, our approach offers a balanced solution. By performing top-*k* sampling on robustly learned coarse tokens, we generate diverse structural scaffolds and subsequently ensure physical stability through a refinement process that progressively predicts fine tokens from these flexible scaffolds.

Specifically, while the coarse stage establishes the macroscopic backbone and introduces stochasticity to ensure broad structural diversity, the fine stage focuses on reconstructing local atomic geometries. Even if the overall backbone is imperfectly formed during the coarse stage, the fine model—conditioned on the sequence—actively corrects these deviations by refining them into physically valid structural tokens, thereby guaranteeing ultimate structural validity.

In conclusion, this work presents a new paradigm that moves beyond static prediction or the sacrifice of stability, leveraging structural hierarchy to generate physically valid yet dynamic conformations. We anticipate that this approach will offer practical and realistic solutions for protein engineering and drug discovery, particularly in tasks requiring the consideration of multiple target states or the de novo design of proteins with novel functions.

## Supplementary Material

btag611_Supplementary_Data

## Data Availability

The model checkpoints and pretrained weights supporting the findings of this study are openly available on Zenodo at https://doi.org/10.5281/zenodo.19412545. The source code for the hierarchical protein conformation generation model, including data preprocessing and training scripts, is available on GitHub at https://github.com/vanha9/hierarchical_conformation_generation.
