# Supplementary material for: Hierarchical discrete representations for coarse-to-fine protein conformation generation
Source: Bioinformatics. 2026 Aug 14;42(9):btag611. doi: 10.1093/bioinformatics/btag611 (PMC13529360; doi:10.1093/bioinformatics/btag611)
Supplement: Supplementary file 1 [file btag611_supplementary_data.pdf]

Supplementary Information for "Hierarchical Discrete Representations for Coarse-to-Fine Protein Conformation Generation"

SeokJun On[1], Yujin Jeong[1], Kang-Hyeon Kim[1], Kyungheon Kang[1], and Eun-Sol Kim[1,2,*]

[1]Department of Artificial Intelligence, Hanyang University, Seoul, Republic of Korea
[2]Department of Computer Science, Hanyang University, Seoul, Republic of Korea
[*]Corresponding author: eunsolkim@hanyang.ac.kr

A. Details on Dataset

Training Dataset

In this work, we trained our protein structure generation model using static protein structures obtained from the Protein Data Bank (PDB) (wwPDB consortium, 2018). The PDB is a curated repository that stores protein sequence information and corresponding three-dimensional structures, including atom types and their coordinates.

To train efficiently within the practical constraints of academic computational resources, our procedure was as follows:

A. Downloaded the full mmCIF dataset without initial filtering from wwPDB.

B. Restricted the data to proteins with a sequence length of 256 or fewer residues.

C. Randomly sampled 24,000 structures to train the tokenization module, and 120,000 structures for the structure language model.

We curated specific datasets for each stage of our framework:

- VQ-VAE Tokenizer Training: For the discretization stage, we utilized a subset of approximately 24,000 structures to train the VQ-VAE codebook.
- Language Model Training: For the subsequent masked token prediction task and coarse-to-fine refinement, we scaled up the dataset to approximately 120,000 structures to capture broader structural diversity and semantic dependencies.

In both stages, the datasets were split into training and validation sets at an 8:2 ratio.

Evaluation Dataset

MD simulation trajectory

Molecular Dynamics (MD) simulation data extends beyond static protein structures, providing continuous trajectory data that captures atomic-level motions and structural changes over time. This data offers diverse conformational ensembles accessible to proteins and is utilized to explore stable states on the energy landscape.

- **Bovine Pancreatic Trypsin Inhibitor (BPTI) (Grouazel et al., 2006)**: BPTI protein serves as a standard model system that has been extensively studied, owing to its small size (58 residues) and high stability. The dataset consists of approximately 420,000 frames derived from a long Molecular Dynamics (MD) simulation conducted at 300 K for a duration of 1 ms (Shaw et al., 2010). Rather than relying solely on geometric similarity, the trajectory was partitioned into five major clusters using a kinetic clustering approach that reflects the protein's dynamical properties. The dynamics of BPTI exhibit a distinct separation of time scales between fast intra-state fluctuations and slow inter-state transitions. Consequently, five thermodynamically stable, long-lived states were defined based on the transition path times and the lifetime of each metastable state.

Conformational-changing pairs

To evaluate the model's capability in capturing structural flexibility and stability, we utilized a dataset of protein pairs that share identical sequences but adopt distinct 3D conformations. These proteins exhibit structural transitions driven by ligand binding or environmental factors, serving as a benchmark to assess the ability to generate multiple conformations beyond single-structure prediction. The dataset is categorized into two subsets based on the mechanism of structural change.

- **Apo/holo** (Saldaño et al., 2022): This subset is constructed to evaluate ligand-induced conformational-changes. It consists of paired structures for the same protein: the Apo state (unbound) and the Holo state (ligand-bound). After excluding sequences that overlap with the training set, we curated 87 pairs for analysis.

- **Fold-switch** (Chakravarty and Porter, 2022): This subset represents heterogeneous proteins capable of undergoing drastic conformational-changes, transitioning between two or more stable secondary or tertiary structures rather than mere local loop fluctuations. These pairs consist of identical sequences that have been experimentally resolved into distinct global folds. After removing sequence overlaps with the training data, 69 pairs were selected for analysis.

Intrinsically disordered proteins

Unlike folded proteins in the Protein Data Bank (PDB), which typically adopt a single, well-defined three-dimensional structure, intrinsically disordered proteins (IDPs) do not possess a fixed conformation and instead exist as a highly dynamic ensemble of diverse structural states.

- **Protein Ensemble Database** (Ghafouri et al., 2023): The Protein Ensemble Database (PED) is a public repository that provides such structural ensembles for both IDPs and proteins containing intrinsically disordered regions. After removing sequence overlaps with the training data, 110 ensembles were selected for analysis.

B. Evaluation Metrics

Structural Accuracy

To assess how close the generated structures are to the corresponding ground truth structures, we employ standard metrics.

- **Root Mean Square Deviation (RMSD)**: The RMSD is the most universally used metric for structural comparison, representing the average distance error between corresponding atoms after two structures are aligned.

$$\text{RMSD} = \sqrt{\frac{1}{N}\sum_{i=1}^{N}\|x_i^{\text{GT}} - x_i^{\text{Pred}}\|^2}$$

  where N is the number of residues, x_i^{GT} and x_i^{Pred} are the coordinates of the i-th Cα atom in the ground truth structure and the predicted structure, respectively.

- **Template Modeling score (TM-score)** : The TM-score measures the similarity of the global topology between two structures (Zhang and Skolnick, 2004).

$$\text{TM-score} = \frac{1}{L_{\text{target}}}\sum_{i=1}^{L_{\text{aligned}}}\frac{1}{1+(\frac{d_i}{d_0(L_{\text{target}})})^2}$$

where L_{target} and L_{aligned} are the numbers of residues in the target protein, and the aligned protein, respectively. The variable d_i denotes the distance between the i-th pair of aligned residues, and $d_0(L_{\text{target}}) = 1.24\sqrt[3]{L_{\text{target}} - 15} - 1.8$ is a scaling factor that normalizes the score to be independent of protein size. The TM-score ranges from 0 to 1, where a higher value indicates better topological similarity.

- **Ensemble score (RMSD-ens, TM-ens)** : These metrics quantify the fidelity and coverage of the generated ensemble by measuring how closely the generated samples approximate the ground truth reference ensemble. Specifically, for each reference structure, we identify the best-matching generated conformation $g \in G$ and average the scores across the entire reference set R.

$$\text{RMSD-ens} = \frac{1}{|R|} \sum_{r \in R} \min_{g \in G} \text{RMSD}(r, g) \tag{S1}$$

$$\text{TM-ens} = \frac{1}{|R|} \sum_{r \in R} \max_{g \in G} \text{TM-score}(r, g) \tag{S2}$$

Physical and Dynamical Properties

- **Pairwise distance (PwD)** : The PwD represents the collection of Euclidean distances between all pairs of residues within a protein. This metric effectively captures the three-dimensional conformation of the protein, allowing it to be represented as a distance matrix or distribution. The distance D_{ij} between the i-th and j-th residues is defined as

$$D_{ij} = \|r_i - r_j\| \quad (1 \leq i, j \leq N)$$

where N is the number of residues, r_i and r_j are the coordinates of the i-th and j-th residues, respectively.

- **Radius of Gyration (RG)** : The RG serves as a metric to quantify the compactness of a protein by measuring how its structural elements are distributed relative to the center of mass. A smaller RG value indicates a tightly packed (folded) structure, whereas a larger value suggests an unfolded or disordered state.

$$\text{RG} = \sqrt{\frac{1}{N} \sum_{i=1}^{N} \|r_i - r_{\text{cm}}\|^2}$$

where N is the number of residues, r_i is the coordinate of the i-th residue, and r_{cm} represents the center of mass of the protein, calculated as $\frac{1}{N}\sum_{i=1}^{N} r_i$

- **Contact map** : The contact map is an $N \times N$ binary matrix that represents the spatial proximity of amino acid residues within a protein. A contact is defined if the distance between two residues falls below a specific threshold. In this metric, self-contacts and contacts between sequence-adjacent residues are disregarded as trivial.

$$C_{ij} = \begin{cases} 1, & \text{if } \|\mathbf{r}_i - \mathbf{r}_j\| < \theta \text{ and } |i - j| > 1 \\ 0, & \text{otherwise} \end{cases}$$

where θ is the distance threshold, defined as 8 Å to capture potential inter-residue interactions. The condition $|i - j| > 1$ filters out trivial backbone connections.

- **Validity** : Validity serves as a metric to evaluate the physical plausibility of generated protein structures by assessing the absence of steric clashes. Based on pairwise distances between C_α atoms, atoms must not be positioned too closely to one another. Validity is calculated as the proportion of structures in the generated ensemble that contain zero clashes.

- **Time-lagged Independent Component (TIC)** : TIC analysis is a dimensionality reduction technique that projects high-dimensional protein coordinate data onto a low-dimensional subspace that best captures slow conformational dynamics (Pérez-Hernández et al., 2013). In contrast to Principal Component Analysis (PCA), which simply maximizes data variance, TIC incorporates a time lag to extract components that maximize the autocorrelation time. This approach effectively filters out high-frequency noise, such as rapid atomic vibrations, and facilitates the identification of significant state transitions, including protein folding and large-scale structural changes.

Statistical Evaluation

- **Jensen-Shannon (JS) divergence** : To measure the distributional discrepancy between the reference and generated ensembles, particularly for the BPTI MD trajectories, we use the JS divergence of PwD, RG, and TIC. JS divergence is a symmetric and smoothed version of the Kullback-Leibler divergence, bounded between 0 and 1.

$$\mathrm{JS}(P\|Q) = \frac{1}{2}\{D_{\mathrm{KL}}(P\|M) + D_{\mathrm{KL}}(Q\|M)\}$$

  where P and Q denote the probability distributions of the reference and generated ensembles, respectively. $M = \frac{1}{2}(P+Q)$ is the average distribution, and $D_{\mathrm{KL}}(P\|Q) = \sum_i P(i)\log_2 \frac{P(i)}{Q(i)}$ is the Kullback-Leibler divergence. A lower JS divergence indicates that the generated distribution closely matches the reference.

- **Mean absolute error (MAE) on ensemble averages** : To evaluate the accuracy of the averaged properties, we compute the Mean Absolute Error (MAE) between the mean values of the generated ensemble and the reference ensemble for PwD, RG, and contact maps.

$$\mathrm{MAE} = \frac{1}{K}\sum_{k=1}^{K} |y_k^{\mathrm{gen}} - y_k^{\mathrm{ref}}|$$

  where K represents the number of features or the dimensionality, y_k^{gen} and y_k^{ref} represents the average values of the k-th feature across the generated and reference ensembles, respectively.

Residue Flexibility

- **Residue flexibility (ResFlex)** : To assess the model's ability to capture position-dependent structural variations, we computed the Pearson correlation between the residue-wise flexibility profiles of the ground truth and the generated ensembles. For datasets with dual reference states (e.g., Apo/holo or fold-switching proteins), the ground truth flexibility was defined by the per-residue distance between the aligned reference pairs. This was compared to the model's flexibility, estimated by the average pairwise distance of aligned generated structures. A high correlation signifies that the model successfully reproduces the native flexibility patterns, exhibiting high variance in flexible regions and stability in rigid regions. We report both Global (gl.) correlations, computed over all residues in the dataset, and Per-target (pt.) correlations, calculated individually for each protein target, and summarized by the mean and median values.

C. Experimental details

Evaluation Setting

To evaluate the performance of our proposed framework, we employed both 3-stage (Coarse→Mid→Fine) and 2-stage (Coarse→Fine) models. Regarding the sampling strategy, we generated samples using greedy sampling and three variations of top-3 sampling with probability ratios of 5:3:2, 4:3:3, and 2:3:5, respectively.

The specific evaluation metrics for each dataset are as follows:

BPTI Dataset: We generated 100 samples for each sampling strategy. For these samples, we identified five kinetic clusters and measured RMSD, RMSD-ens, and TM-ens. Furthermore, we compared the generated samples against 420,000 frames of MD simulation data by calculating the JS-divergence for physical property distributions, including PwD, RG and TIC. Structural integrity was also assessed via clash validity.

Conformational-changing Pairs (Apo/holo and Fold-switch): We generated 5 samples for each sampling strategy. To evaluate the model's ability to capture structural transitions, we utilized ResFlex and TM-score as primary metrics.

Intrinsically Disordered Proteins (PED Dataset): We generated 10 samples for each sampling strategy. For PED, we calculated the MAE for various biophysical properties, such as PwD, RG, and contact maps, to measure the discrepancy between the generated ensembles and the experimental ground truth.

Experimental Results

In this section, we present supplementary experimental results and qualitative analyses. In the following tables, **bold** and underlined values indicate the best and second-best results for each metric, respectively.

Tables S1 to S4 report detailed evaluation results on the BPTI molecular dynamics trajectory, conformational-changing pairs, and the Protein Ensemble Database (PED). Fig. S1 provides a qualitative comparison for long-sequence proteins. It highlights instances where single-resolution baselines fail to maintain global alignment or generate valid secondary structures, whereas our hierarchical approach successfully reconstructs these features. Finally, Table S5 presents an ablation study applying our proposed stochastic top-k sampling strategy to the single-resolution baseline (ESMDiff) to assess the impact of the sampling method on performance.

Table S1: Performance comparison of 2-stage and 3-stage models across different sampling strategies on five BPTI kinetic clusters (C1–C5). Performance is evaluated using RMSD, where lower values indicate better structural accuracy (↓).

Method		RMSD_{C1}	RMSD_{C2}	RMSD_{C3}	RMSD_{C4}	RMSD_{C5}
MSA-Subs.		0.921	1.745	2.519	1.688	0.989
AlphaFlow		0.939	1.683	2.394	1.401	0.980
ESMFlow		0.915	1.694	2.424	<u>1.391</u>	0.988
Str2Str (PF)		2.092	2.082	2.774	2.310	2.078
Str2Str (SDE)		2.607	2.457	3.068	2.767	2.431
EigenFold		0.829	1.676	2.470	1.436	1.009
ESMDiff (ID)		0.928	1.634	2.278	1.507	1.010
ESMDiff (DDPM)		0.939	1.620	2.208	1.405	1.001
Ours (3-stage)	Greedy sampling	<u>0.828</u>	1.691	2.416	1.430	<u>0.923</u>
	Top-3 (5:3:2)	0.937	1.730	**2.083**	1.478	1.006
	Top-3 (4:3:3)	1.013	1.622	2.153	1.602	0.963
	Top-3 (2:3:5)	1.027	1.683	<u>2.102</u>	1.492	1.154
Ours (2-stage)	Greedy sampling	**0.798**	1.671	2.344	**1.345**	**0.888**
	Top-3 (5:3:2)	0.845	1.618	2.299	1.559	0.975
	Top-3 (4:3:3)	0.940	<u>1.607</u>	2.183	1.545	0.938
	Top-3 (2:3:5)	1.032	**1.571**	2.130	1.735	0.951

Table S2: Evaluation of physical properties and ensemble quality using BPTI MD trajectories. Metrics include JS divergence, validity, RMSD-ens, and TM-ens. Lower values for JS divergence and RMSD-ens(↓), and higher values for TM-ens and validity(↑), indicate better performance.

Method		Distribution Divergence			Structural Fidelity		
		JS-PwD	JS-TIC	JS-RG	Validity	TM-ens	RMSD-ens
MSA-Subs.		0.615	0.499	0.730	<u>0.990</u>	0.835	1.576
AlphaFlow		0.474	0.499	0.730	**1.000**	0.843	1.479
ESMFlow		0.488	0.487	0.734	0.980	0.839	1.482
Str2Str(PF)		0.503	0.513	0.326	0.910	0.705	2.267
Str2Str(SDE)		0.531	0.570	0.394	0.860	0.651	2.666
EigenFold		0.512	0.487	0.768	0.660	0.839	1.482
ESMDiff(ID)		0.378	0.358	0.452	0.950	0.844	1.471
ESMDiff(DDPM)		0.346	<u>0.341</u>	0.356	0.940	0.845	<u>1.435</u>
Ours (3-stage)	Greedy sampling	0.452	0.370	0.457	0.920	0.842	1.458
	Top-3 (5:3:2)	0.339	0.422	0.283	0.950	0.836	1.447
	Top-3 (4:3:3)	<u>0.330</u>	0.450	0.278	0.950	0.838	1.467
	Top-3 (2:3:5)	0.336	0.459	0.153	0.960	0.830	1.491
Ours (2-stage)	Greedy sampling	0.454	**0.281**	0.356	<u>0.990</u>	**0.850**	**1.409**
	Top-3 (5:3:2)	0.335	0.390	0.196	**1.000**	<u>0.847</u>	1.459
	Top-3 (4:3:3)	**0.323**	0.401	<u>0.136</u>	<u>0.990</u>	0.841	1.443
	Top-3 (2:3:5)	0.336	0.402	**0.107**	<u>0.990</u>	0.837	1.484

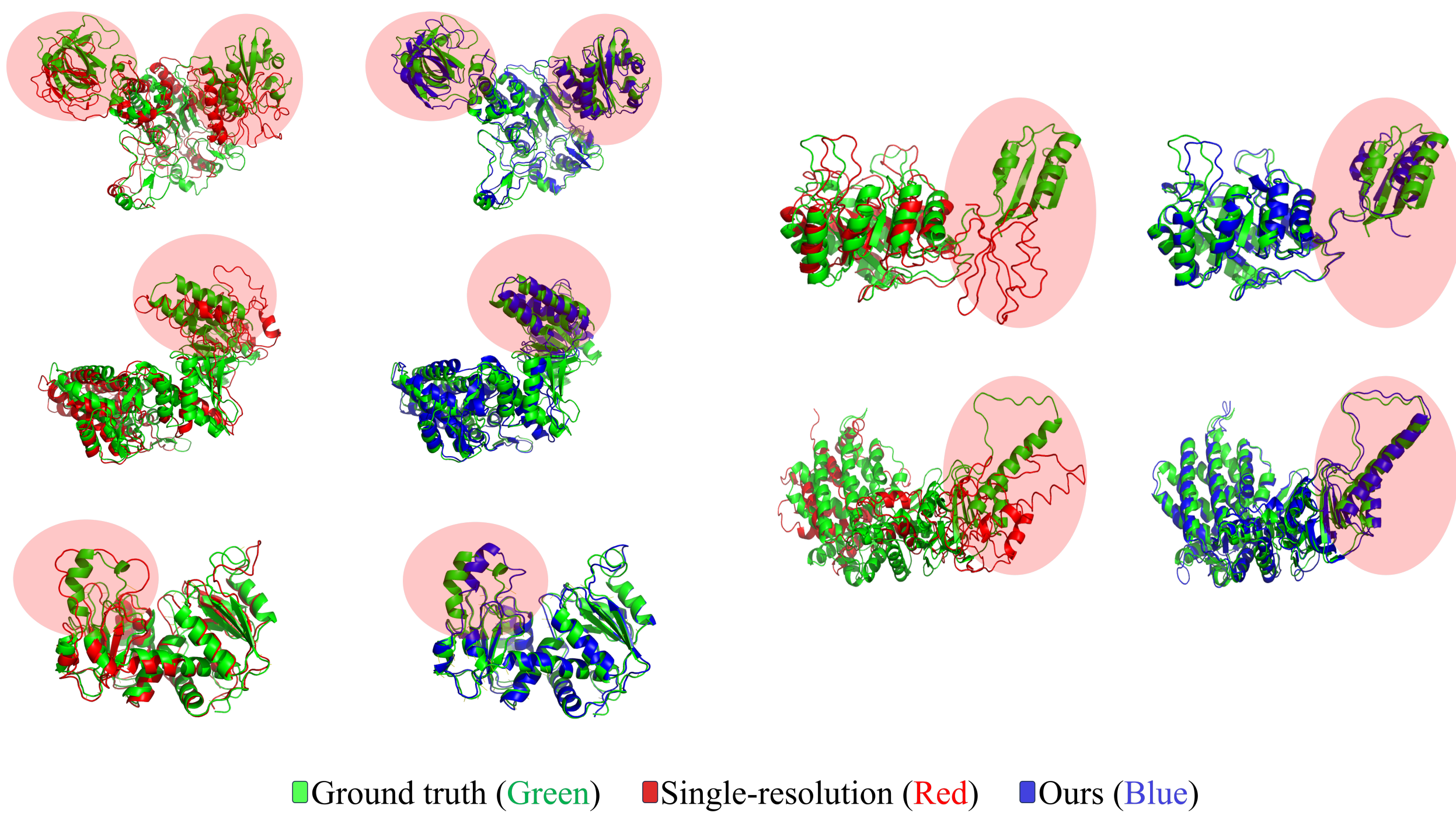

Figure S1: **Extended comparison of structural fidelity in challenging regions.** This figure presents additional qualitative examples demonstrating the performance gap in complex local structures (pink circles). Consistent with the main text, the single-resolution model (Red) exhibits structural deviations, whereas our method (Blue) accurately reconstructs the native topology, aligning closely with the ground truth (Green).

Table S3: Evaluation of ResFlex and TM-ens on conformational-changing pairs. We report the mean and median values for ResFlex pt. and TM-ens. Higher values for both metrics indicate improved performance(↑).

Method		Apo/holo			Fold-switch		
		ResFlex gl.	ResFlex pt.	TM-ens	ResFlex gl.	ResFlex pt.	TM-ens
MSA-Subs.		0.445	0.422 / 0.382	**0.863** / **0.895**	0.262	0.304 / 0.318	**0.739** / **0.814**
AlphaFlow		**0.563**	**0.533** / **0.565**	<u>0.862</u> / 0.885	0.394	**0.381** / **0.373**	<u>0.734</u> / <u>0.812</u>
ESMFlow		0.448	<u>0.506</u> / <u>0.523</u>	0.854 / <u>0.889</u>	0.327	0.343 / 0.351	0.702 / 0.758
Str2Str(PF)		0.136	0.314 / 0.326	0.744 / 0.763	0.150	0.203 / 0.177	0.598 / 0.629
Str2Str(SDE)		0.061	0.294 / 0.284	0.578 / 0.585	0.048	0.177 / 0.184	0.431 / 0.419
EigenFold		0.121	0.404 / 0.401	0.826 / 0.869	0.328	0.272 / 0.222	0.617 / 0.659
ESMDiff(ID)		0.458	0.488 / 0.507	0.842 / 0.877	0.391	0.323 / 0.352	0.657 / 0.719
ESMDiff(DDPM)		0.373	0.478 / 0.488	0.841 / 0.884	0.408	0.331 / 0.341	0.635 / 0.706
Ours (3-stage)	Greedy sampling	0.437	0.493 / 0.521	0.844 / 0.876	0.410	0.313 / 0.320	0.631 / 0.726
	Top-3 (5:3:2)	<u>0.486</u>	0.464 / 0.484	0.839 / 0.880	0.433	0.326 / 0.329	0.626 / 0.701
	Top-3 (4:3:3)	0.449	0.466 / 0.495	0.831 / 0.865	**0.453**	<u>0.345</u> / <u>0.355</u>	0.629 / 0.702
	Top-3 (2:3:5)	0.422	0.432 / 0.410	0.824 / 0.865	0.387	0.326 / 0.316	0.622 / 0.711
Ours (2-stage)	Greedy sampling	0.419	0.480 / 0.453	0.854 / 0.887	<u>0.447</u>	0.305 / 0.290	0.642 / 0.716
	Top-3 (5:3:2)	0.318	0.496 / 0.514	0.847 / 0.882	0.410	0.309 / 0.307	0.643 / 0.714
	Top-3 (4:3:3)	0.365	0.482 / 0.518	0.840 / 0.872	0.396	0.305 / 0.304	0.640 / 0.710
	Top-3 (2:3:5)	0.309	0.458 / 0.450	0.837 / 0.862	0.419	0.302 / 0.335	0.632 / 0.703

Table S4: Performance evaluation on the Protein Ensemble Database (PED), measuring the MAE of physical properties. We report the mean and median values. Lower MAE values indicate better performance(↓).

Method		Pairwise distance	Radius of Gyration	Contact map
MSA-Subs		6.691 / <u>4.214</u>	4.245 / 2.213	**0.178** / **0.112**
AlphaFlow		7.161 / **3.553**	4.915 / **1.562**	0.231 / 0.162
ESMFlow		7.622 / 4.215	5.043 / <u>1.592</u>	0.259 / 0.189
Str2Str(PF)		8.717 / 5.626	5.280 / 2.834	0.266 / 0.192
Str2Str(SDE)		10.222 / 7.769	6.271 / 3.796	0.313 / 0.235
EigenFold		12.268 / 6.127	9.267 / 4.632	1.316 / 0.609
ESMDiff(ID)		6.953 / 4.945	4.506 / 2.337	0.289 / 0.211
ESMDiff(DDPM)		7.085 / 4.831	4.531 / 2.204	0.352 / 0.266
Ours (3-stage)	Greedy sampling	<u>6.667</u> / 4.633	<u>4.093</u> / 2.229	0.267 / 0.212
	Top-3 (5:3:2)	7.962 / 5.334	5.116 / 2.794	0.211 / 0.144
	Top-3 (4:3:3)	7.592 / 5.263	4.652 / 2.200	0.211 / 0.152
	Top-3 (2:3:5)	7.599 / 5.075	4.604 / 1.924	0.218 / 0.151
Ours (2-stage)	Greedy sampling	**6.493** / 4.879	**4.058** / 2.198	0.247 / 0.178
	Top-3 (5:3:2)	7.965 / 5.065	5.135 / 2.240	<u>0.201</u> / <u>0.133</u>
	Top-3 (4:3:3)	7.965 / 5.143	5.186 / 2.898	0.205 / 0.135
	Top-3 (2:3:5)	7.586 / 5.202	4.826 / 2.685	0.207 / 0.140

Table S5: Performance comparison of our proposed method (hierarchical approach) and stochastic top-k sampling on ESMDiff (single resolution)

Method		$RMSD_{C1}$	$RMSD_{C2}$	$RMSD_{C3}$	$RMSD_{C4}$	$RMSD_{C5}$
ESMDiff	Greedy sampling	0.939	1.620	2.208	<u>1.405</u>	1.001
	Top-3 (5:3:2)	0.897	1.628	2.379	1.621	<u>0.907</u>
	Top-3 (4:3:3)	0.888	1.615	2.364	1.505	0.927
	Top-3 (2:3:5)	0.866	1.625	2.342	1.639	0.926
Ours (3-stage)	Greedy sampling	<u>0.828</u>	1.691	2.416	1.430	0.923
	Top-3 (5:3:2)	0.937	1.730	**2.083**	1.478	1.006
	Top-3 (4:3:3)	1.013	1.622	2.153	1.602	0.963
	Top-3 (2:3:5)	1.027	1.683	<u>2.102</u>	1.492	1.154
Ours (2-stage)	Greedy sampling	**0.798**	1.671	2.344	**1.345**	**0.888**
	Top-3 (5:3:2)	0.845	1.618	2.299	1.559	0.975
	Top-3 (4:3:3)	0.940	<u>1.607</u>	2.183	1.545	0.938
	Top-3 (2:3:5)	1.032	**1.571**	2.130	1.735	0.951

References

Chakravarty D and Porter L. L. Alphafold2 fails to predict protein fold switching. *Protein Science* 2022;31:e4353.

Ghafouri H et al. Ped in 2024: improving the community deposition of structural ensembles for intrinsically disordered proteins. *Nucleic Acids Research* 2023;52:D536–44.

Grouazel S et al. Exploring bovine pancreatic trypsin inhibitor phase transitions. *J. Phys. Chem. B* 2006;110:19664–70.

Pérez-Hernández G et al. Identification of slow molecular order parameters for markov model construction. *The Journal of Chemical Physics* 2013;139:015102.

Saldaño T et al. Impact of protein conformational diversity on alphafold predictions. *Bioinformatics* 2022;38:2742–48.

Shaw D. E et al. Atomic-level characterization of the structural dynamics of proteins. *Science* 2010;330:341–46.

wwPDB consortium. Protein data bank: the single global archive for 3d macromolecular structure data. *Nucleic Acids Research* 2018;47:D520–28.

Zhang Y and Skolnick J. Scoring function for automated assessment of protein structure template quality. *Proteins: Structure, Function, and Bioinformatics* 2004;57:702–10.
